# Sultiame pharmacokinetic profile in plasma and erythrocytes after single oral doses: A pilot study in healthy volunteers

**DOI:** 10.1002/prp2.558

**Published:** 2020-01-28

**Authors:** Kim Dao, Paul Thoueille, Laurent A. Decosterd, Thomas Mercier, Monia Guidi, Carine Bardinet, Sébastien Lebon, Eva Choong, Arnaud Castang, Catherine Guittet, Luc‐André Granier, Thierry Buclin

**Affiliations:** ^1^ Service of Clinical Pharmacology Lausanne University Hospital University of Lausanne Lausanne Switzerland; ^2^ School of Pharmaceutical Sciences Institute of Pharmaceutical Sciences of western Switzerland Geneva Switzerland; ^3^ Unit of Paediatric Neurology and Neurorehabilitation Department Mother‐Woman‐Child Lausanne University Hospital University of Lausanne Lausanne Switzerland; ^4^ Advicenne Pharma SA Nîmes France

**Keywords:** clearance, healthy volunteers, pharmacokinetic, sultiame, volume of distribution

## Abstract

A pilot study was conducted aiming at specifying sultiame's pharmacokinetic profile, completed by in vitro assays evaluating the intraerythrocytic transfer of sultiame and by a pharmacokinetic model assessing its distribution. Single oral doses of sultiame (Ospolot^®^ 50, 100, and 200 mg) were administered in open‐label to four healthy volunteers. Serial plasma, whole blood, and urine samples were collected. A spiking experiment was also performed to characterize sultiame's exchanges between plasma and erythrocytes in vitro. Pharmacokinetic parameters were evaluated using standard noncompartmental calculations and nonlinear mixed‐effect modeling. The plasma maximal concentrations (*C*_max_) showed striking nonlinear disposition of sultiame, with a 10‐fold increase while doses were doubled. Conversely, whole blood C_max_ increased less than dose proportionally while staying much higher than in plasma. Quick uptake of sultiame into erythrocytes observed in vivo was confirmed in vitro, with minimal efflux. A two‐compartment model with first‐order absorption, incorporating a saturable ligand to receptor binding, described the data remarkably well, indicating apparent plasma clearance of 10.0 L/h (BSV: 29%) and distribution volume of 64.8 L; saturable uptake into an intracellular compartment of 3.3 L with a maximum binding capacity of 111 mg accounted for nonlinearities observed in plasma and whole blood concentrations. Pharmacokinetic characteristics of sultiame are reported, including estimates of clearance and volume of distribution that were so far unpublished. The noticeable nonlinearity in sultiame disposition should be taken into account for the design of future studies and the interpretation of therapeutic drug monitoring results.

**Abbreviations**
AUC: area under the curve
BSV: between subject variability
CA: carbonic anhydrase
CL/F: apparent clearance
C_max_: plasma maximal concentrations
F: bioavailability
IS: internal standard
meOH: methanol
MW: molecular weight
PK: pharmacokinetic
TSQ: triple quadrupole mass spectrometer
VAS: visual analog scales
V/F: apparent volume of distribution

## 1 INTRODUCTION

Sultiame (Bayer 1960, MW 290 g/mol)^1^ is a cyclic sulfonamide, whose antiepileptic activity is thought to mainly result from the inhibition of various subtypes of carbonic anhydrase (CA).^2^ In particular, the blockade of cytosolic CA II seems to produce a degree of intracellular acidification sufficient to stabilize seizure‐eliciting neurons. In addition, an inhibitory action on sodium channels possibly contributes to its antiepileptic efficacy.^2^

Sultiame is a first choice treatment in some countries for benign childhood epilepsy with centrotemporal spikes, a common nonlesional epilepsy syndrome of childhood usually appearing between 4 and 12 years of age characterized by nocturnal oro‐facial motor or sensory seizures, with or without bilateral propagation.^3^ The overall prognosis is good, with spontaneous disappearance of epilepsy at adolescence. However, some children may have mild neurocognitive deficits sometimes linked to interictal epileptiform discharges on electroencephalogram. Although not all cases deserve a treatment, patients with more frequent seizures benefit from a course of antiseizure drug. Sultiame has been reported to be well tolerated and possibly more effective than other antiepileptic drugs commonly used in focal seizures, such as carbamazepine, gabapentine, or levetiracetam.^3^, ^4^, ^5^

Despite a regular use over decades by pediatric neurologists, the pharmacokinetic (PK) profile of sultiame was only scarcely studied in humans.^6^, ^7^, ^8^, ^9^ Linear disposition is assumed by the manufacturer, with a half‐life described to lie “between 2 and 16 hours”, while no values have been published for bioavailability (F), apparent volume of distribution (V/F), and apparent clearance (CL/F). Plasma‐free fraction amounts to 71%. Plasma levels are said to display a significant degree of between‐ and within‐individual variability, even at steady state. The manufacturer mentions a mixed elimination by metabolism (hydroxylation) and renal excretion, the latter concerning 30%‐60% of an ingested dose. Two metabolites of sultiame have been identified in urines, the main one being hydroxy‐sultiame (an inactive compound).^8^

Our primary aim was to assess sultiame's PK parameters, in order to better characterize its concentration‐time profile. Preliminary investigations of the stability of blood samples for PK measurement indicated a marked uptake into erythrocytes that we possibly attributed to sultiame's affinity for CA abundant in erythrocytes.^10^ A secondary aim was thus defined to characterize sultiame's exchanges between plasma and erythrocytes through an in vitro spiking experiment.

## 2 MATERIAL AND METHODS

### 2.1 Subjects

Eligible subjects for the clinical study were healthy adult male volunteers aged 18‐45 years, with a body weight ranging between 55 and 95 kg and a body mass index of 18‐29 kg/m^2^. Subjects with a history or evidence of clinically significant diseases or interfering conditions were excluded. Other exclusion criteria included: history of any clinically significant laboratory value including serology for hepatitis and HIV, relevant alcohol or drug abuse, recent acute illness, or use of any medication the week prior to study.

Single oral doses of 50, 100, and 200 mg of sultiame (Ospolot^®^, immediate‐release coated tablets) were administered in fasting conditions, in open‐label, during periods 1, 2, and 3, respectively, at 4‐6 weeks interval. On each period, serial plasma and whole blood samples were collected predose, then at 15, 30 min, 1, 2, 3, 4, 5, 6, 8, 10, 24, 48, 72 168, 336, and 504 hours postdose. Plasma and whole blood samples were stored at − 80°C until analysis. Hematocrit was measured using a microhematocrit centrifuge machine (Haematokrit 200^®^, Hettich AG, Bäch, Switzerland) on each occasion to calculate intraerythrocytic concentrations. Urine PK samples were collected in 6 fractions, before dosing, from 0‐2, 2‐4, 4‐8, 8‐10, and 10‐24 hours postdose. Urine pH was measured on each fraction.

The study protocol had been submitted to and approved by the competent research ethics committee (CER‐VD Nr 2017‐02005) and regulatory authority (Swissmedic Nr 2017DR1188; Clinicaltrials.gov identifier: NCT03400189).

### 2.2 Analytical method

The quantification of sultiame in whole blood, plasma, and urine was performed using a high‐performance liquid chromatography coupled to tandem triple quadrupole mass spectrometry (LC‐MS/MS) method developed and validated purposely for the trial. Briefly, 300 µL of methanol (MeOH) containing the internal standard (IS) sultiame‐D4 (Toronto Research Chemicals, Canada) was added to 100 µL of human plasma, hemolyzed whole blood, or hemolyzed red blood cells, for protein precipitation. Urine samples were processed similarly. After vortex, sonication for 30 seconds, and centrifugation steps, 250 µL of supernatant was transferred into a glass vial with insert, followed by dilution with 250 µL of pure water and a final vortex step, prior to LC‐MS/MS analysis. Chromatographic analyses were carried out on a XSelect HSS T3 column (Waters, Milford, MA, USA) using a water‐acetonitrile gradient over 5 minutes. Detection was performed with a triple quadrupole mass spectrometer (TSQ Quantum Ultra, ThermoFisher Scientific, Waltham, MA, USA) using the *m/z* transitions 289.0 → 225.1 for sultiame, and 293.0 → 229.1 for sultiame‐D4. Lower limits of detection and quantification of the method were 0.001 and 0.01 µg/mL, respectively, with a dynamic range extending up to 50 µg/mL. No qualitative matrix effect was observed at sultiame retention time. Deviations from nominal concentration (inaccuracy) of internal QCs were comprised within − 1.9 to + 11.7% over the calibration range. The developed assay has been applied for the analyses of Certified External QC samples of sultiame in plasma (ClinChek^®^ N° 14,082 and RECIPE Chemicals, Munich, Germany) with deviations from nominal concentrations comprised between − 4.4 to + 3.4%, demonstrating the excellent accuracy of the developed method. Metabolites, whose chemical structure is still elusive, were not determined.

### 2.3 Pharmacokinetic parameters

Erythrocytic concentrations (C_ery_) were deduced from whole blood and plasma measurements as:

$$C_{\text{ery}}=\frac{C_{\text{whole blood}}-C_{\text{plasma}}\cdot \left(1-\text{Ht}\right)}{\text{Ht}}$$

where Ht is the hematocrit of the sample.^11^

#### 2.3.1 Noncompartmental analysis

Plasma, whole blood, and urine sultiame PK parameters were first computed for each volunteer using standard noncompartmental calculations with Stata^®^ (version 13, StataCorp. 2013, Stata Statistical Software, College Station TX, USA).

The area under the curve for a single dose (AUC_0‐inf_) was calculated using the log trapezoidal rule with extrapolation to infinity. The terminal rate constant ($\lambda_{z}$) was derived from a two‐exponential model curve. CL/F was calculated as the dose divided by AUC_0‐inf_, the half‐life (t_1/2_) as ln(2)/$\lambda_{z}$, and V/F as (CL/F)/$\lambda_{z}$.

#### 2.3.2 Compartmental analysis

**Figure 1:**
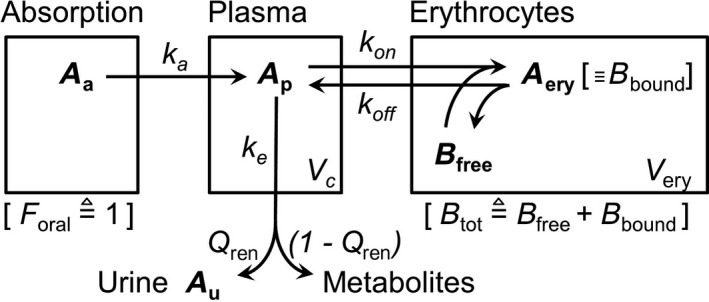
Compartmental model accounting for nonlinearity of distribution. A_a_, amount of sultiame at the absorption site; A_p_, amount of sultiame in plasma and central compartment; V_c_, central volume of distribution; A_ery_, amount of sultiame in the cells, assumed to be bound to receptors; V_ery_, cellular volume of distribution; k_a_, absorption rate constant; k_e_, elimination rate constant; k_on_, constant of association onto receptors binding sites; k_off_, constant of dissociation from the binding site; B_bound_, amount of occupied receptors, assumed equal to A_ery_; B_free_, amount of free binding sites; B_tot_, sultiame maximal binding capacity (mg); Q_ren_, renal fraction of the elimination rate; F_oral_, oral bioavailability, assumed equal to 1

A population PK analysis was also performed using a nonlinear mixed‐effect modeling approach (NONMEM version 7.4, ICON Development Solutions, Hanover MD, USA). Based on graphical exploration and in vitro experiments, a two‐compartment model with first‐order absorption incorporating a saturable ligand to receptor binding was devised, as illustrated in Figure 1, to account for sultiame's affinity for CA abundant in erythrocytes. The model was expressed in terms of differential equations and first‐order rate constants of absorption (k_a_), elimination (k_e_), association with (k_on_) and dissociation from (k_off_) receptors, maximal binding capacity (B_tot_), apparent central volume of distribution (V_c_/F), and erythrocytes’ volume of distribution (V_ery_). Note that V_ery_ actually encompasses the volume of all types of cells containing receptors, assumed to tally with the sampled erythrocytes. Plasma protein binding was assumed constant over the whole range of observed concentrations. Renal extraction fraction (Q_ren_) was added to characterize urine excretion with an additional compartment for urine data. Values of k_on_ and k_off_ characterized in vitro at different temperatures were used as initial estimates and the value of k_on_ to 2018 µM^−1^ h^−1^ at 37°C fixed in the in vivo model. F was not evaluated, making the model estimate apparent values for V_c_/F and CL/F. The first‐order elimination rate constant from the central compartment was calculated as k_e_ = (CL/F)/(V_c_/F). The first value below the LLOQ was equaled to LLOQ/2 and subsequent nonquantifiable values were discarded (M6 method).^12^ Details concerning differential equations and model building steps and adjustment are available in Supplemental material.

A stepwise procedure was used to identify the model that best fitted the data, comparing two‐, three‐, and four‐compartmental models (peripheral or separate intracellular compartments for free and bound sultiame), and other nonlinear models (B_max_ sigmoid model). Exponential errors were used for the description of between‐subject variability (BSV) of PK parameters. Proportional, additive, and mixed error models were compared to describe the residual variability. The stability and internal validity of the final model were assessed by means of standard procedures, detailed in Supplemental material. Covariates were not analyzed due to the limited number of subjects included.

### 2.4 Safety assessments

All adverse events were recorded with an evaluation of their severity and imputability to the study drug. Each volunteer received a full medical examination at screening and at the end of the study. Visual analogue scales (VAS) on paper support recorded the intensity of 12 potential symptoms (abdominal pain, appetite, nausea, taste of sparkling water, fatigue, headache, dyspnea, dizziness, paresthesia, difficulty in concentration, irritability, and depression) predrug (0 hour) at 0.5, 1, 2, 4, 6, 8, 10, and 24 hours postdose. Vital signs (supine blood pressure, heart rate, body temperature, and respiratory frequency) were measured predose and at 1, 2, 4, 6, 8, 10, and 24 hours postdose. Laboratory parameters, drug screen, and urinalysis were performed at screening and at study end.

### 2.5 In vitro assays

A spiking experiment was performed to better characterize sultiame exchanges between plasma and erythrocytes. The influx into cells was investigated through spiking fresh human whole blood on EDTA at different target concentrations (1, 5, 10, 20, 40, and 80 mg/L) and different temperatures (4, 21, and 37°C), followed by sampling at 0, 2, 4, 6, 8, 10, 15, 30, 60, 120, and 180 min. The efflux was investigated through incubating whole blood for 1 h at 2 concentrations (10 and 40 mg/L) at 37°C, centrifugating and briefly washing the erythrocytes with NaCl 0.9%, and letting them equilibrate with fresh plasma while drawing samples at 0, 2, 4, 6, 8, 10, 15, 30, 60, 120, 180 minutes, and 24 hours.

In addition, competition studies were performed with two other antiepileptic agents known for their affinity for CA (zonisamide and topiramate), added at equimolar concentration to 20 mg/L of sultiame. Similar ranges of inhibition constants for CA II were reported in the literature for these three substances.^13^ Samples (1 mL) were drawn before and at 2, 4, 6, 8, 10, 15, 20, 30, 45, 60, and 120 min after addition of the competitor. Sultiame, zonisamide, and topiramate concentrations were then analyzed. All samples were centrifuged at 18 620 *g* for 1 minutes at 4°C. Plasma, erythrocyte, and urine fractions were stored separately at − 80°C prior to analysis for a maximum of 9 months. For analysis, plasma and hemolyzed erythrocytes samples were subjected to protein precipitation with MeOH containing the internal‐standard sultiame D4 and quantified with the previously described HPLC‐MS/MS method, using matrix‐matched calibration and quality controls. The sultiame concentrations determined in erythrocytes were corrected for the 10% interstitial plasma remnant in the erythrocyte fraction after centrifugation.^14^

## 3 RESULTS

### 3.1 Subjects

Four male subjects completed this study part without any drop‐out. Age (mean ± SD) was 25 ± 3 years (range 22‐28 years) and weight 69 ± 2 kg (range 65‐71 kg) at inclusion. Safety and tolerability assessments revealed limited impact on gastrointestinal and central nervous system tolerance using VAS. Fatigue was the symptom most frequently reported. Transient facial and limb paresthesia were reported by three subjects after intake of the highest dose of 200 mg. No severe or serious adverse events were reported.

### 3.2 Noncompartmental pharmacokinetic parameters

**Figure 2:**
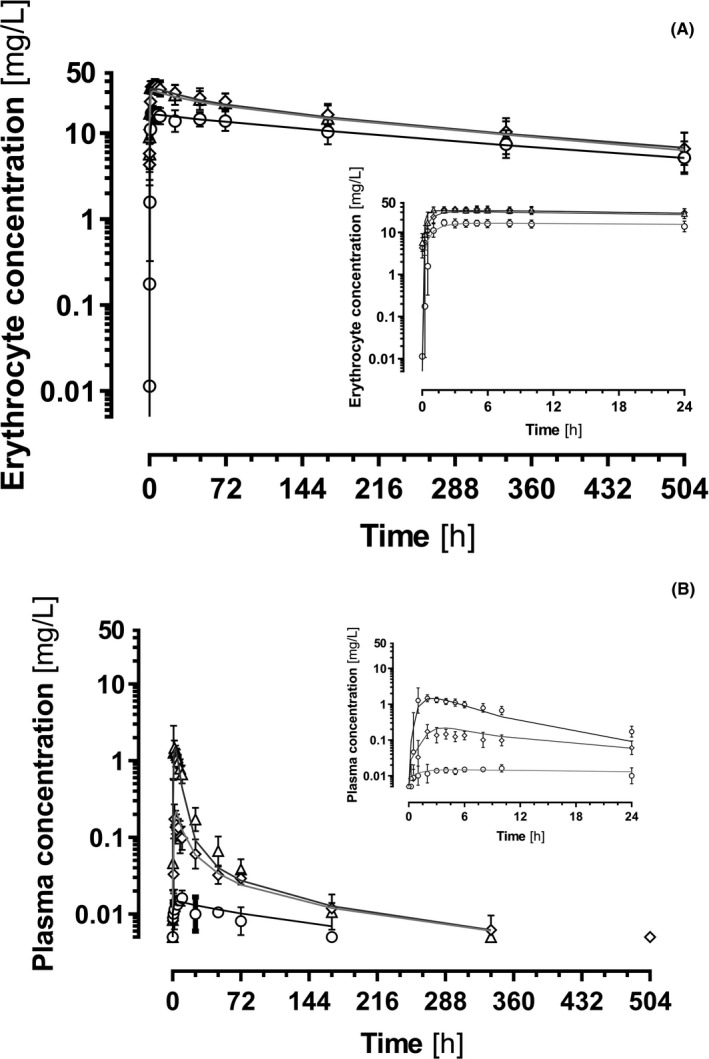
Log‐transformed plasma and erythrocyte concentrations of sultiame: predicted concentration‐time profiles (continuous lines) and geometric mean and standard errors of observations in erythrocytes (A) and plasma (B), after administration of single doses of 50 mg (circles), 100 mg (diamonds), and 200 mg (triangles). Insets zoom on the first 24 hours after drug intake

Average sultiame plasma and whole blood concentrations after single oral doses of 50, 100, and 200 mg, administered at 4 weeks intervals, are represented in Figure 2. The plasma maximal concentration (C_max_) revealed striking nonlinear disposition of sultiame, with 10‐fold increases between each dose levels, while doses only doubled. Conversely, whole blood C_max_ increased less than dose proportionally and were much higher than those in plasma. PK parameters of sultiame obtained by noncompartmental calculations are reported in Table 1 for each dose. Nonlinearity for CL/F and area under the curve (AUC_0‐inf_) is salient between dose levels, suggesting a saturation process in distribution in erythrocytes. Low Vc/F values for whole blood sultiame contrast with high values for plasma, in line with the important intracellular uptake. Whole blood AUC_0‐inf_ values are much higher than plasma AUC_0‐inf_ values and show inverse trends for dose dependency compared to CL/F estimates. A fair degree of interindividual variability is observed between subjects. Nonlinearity is noted as well in the urinary recovery of unchanged sultiame, which increases about 10‐fold between the 50 and 200 mg doses. Still renal CL remains unaffected by dose.

**Table 1:** Noncompartmental pharmacokinetic parameters

	Sultiame 50 mg (n = 4)	Sultiame 100 mg (n = 4)	Sultiame 200 mg (n = 4)
Plasma parameters			
C_max_ (µg/mL)	0.02 (16)	0.18 (44)	1.88 (24)
Median t_max_ (h) (range)	7.0 (2.0‐10.0)	2.0 (2.0‐6.1)	1.5 (1.0‐2.0)
AUC_(0‐inf)_ (µg·h mL^−1^)	1.24 (63)	8.57 (33)	24.04 (27)
t_1/2_ (h)	50.8 (62)	90.9 (19)	40 (31)
CL/F (L/h)	40.4 (93)	11.7 (25)	8.3 (26)
V/F (L)	2964 (21)	1529 (21)	480 (26)
Whole blood parameters			
C_max_ (µg/mL)	7.91 (17)	15.87 (19)	17.19 (17)
Median t_max_ (h) (range)	3.0 (2.0‐8.0)	5.01 (5.0‐6.0)	2.5 (1.0‐3.0)
AUC_(0‐inf)_ (µg·h mL^−1^)	3323 (44)	4535 (39)	4527 (43)
t_1/2_ (h)	313 (23)	233 (16)	253 (21)
CL/F (L/h)	0.015 (37)	0.008 (34)	0.044 (41)
V/F (L)	6.8 (21)	7.4 (21)	16.2 (27)
Urine parameters			
Recovery unchanged (%)	1.61 (17)	5.24 (111)	16.73 (36)
Renal clearance (L/h)	0.17 (30)	0.14 (49)	0.13 (24)

Note

Geometric means (CV%) of noncompartmental plasma and whole blood PK parameters of sultiame after administration of single doses of 50, 100, and 200 mg.

### 3.3 Compartmental analysis

A 2‐compartment model incorporating a saturable ligand to receptor binding, completed with the in vitro k_on_ and k_off_ values and a compartment for urines, best described the data. In addition to CL/F (ΔOFV of − 74), BSV on Q_Ren_ (ΔOFV − 21) was incorporated into the model. Intrapatient residual variability was best described using a proportional error for plasma, erythrocytes, and urine values. A relatively simple ligand to receptor model captured remarkably well the nonlinear kinetics of sultiame across the dose levels, assuming a saturable amount of binding sites in erythrocytes and possibly other types of cells. There were no indications for using more complicated models: for example, a sigmoid B_max_ model did not perform better (ΔAIC + 29); adding a linear component to account for nonsaturable transfer of sultiame into erythrocytes did not provide strong evidence of model improvement (ΔAIC − 2.5). Conversely, classical linear models were unable to describe the data. The final base population parameters are listed in Table 2 and the average concentration predictions for each dose level are shown on Figure 2. Individual profiles of predicted concentrations are available in Supplemental material along with goodness‐of‐fit plots, normalized prediction error (NPDE), and results of pcVPC.

**Table 2:** Final population compartmental model parameters

Parameter	Population pharmacokinetics analysis				Bootstrap evaluation			
	Estimate	RSE(%)	BSV(%)	RSE (%)	Median	CI_95%_	BSV(%)	CI_95%_
CL_plasma_ (L/h)	9.95	11.8	28.8	15	9.95	(8.4;11.8)	27	(0.5;42)
V_c_ (L)	64.8	18.1	–	–	64.8	(52.1;80.6)	–	–
V_ery_ (L)	3.27	7	–	–	3.27	(2.8;3.6)	–	–
k_a_ (h^−1^)	1.88	13.8	–	–	1.88	(1.3;2.1)	–	–
k_on_ (h^−1^·µM^−1^)	2018	–	–	–	2018	–	–	–
k_off_ (h^−1^)	8080	19.2	–	–	8080	(6626;11209)	–	–
B_tot_ (mg)	111	2.1	–	–	111	(105;117)	–	–
Q_Ren_ (%)	28.8	13	24.3	67.6	28.8	(22.9;37.4)	24	(0.8;33)
σ_prop,c_ (CV%)	0.426	9	–	–	0.424	(0.38;0.45)	–	–
σ_prop,ery_ (CV%)	0.3	11	–	–	0.299	(0.21;0.32)	–	–
σ_prop,urine_ (CV%)	0.39	34	–	–	0.39	(0.24;0.49)	–	–

Abbreviations: BSV, Between‐subject variability; B_tot_, ligand maximal binding capacity; CI95%, 95% confidence interval; CL_plasma_, plasma clearance; k_a_, constant of absorption; k_off_, dissociation constant; k_on_, association constant, not estimated but fixed to 2018 h^−1^ µM^‐1^; Q_Ren_, fraction eliminated by renal route; RSE, Relative standard error of the estimate, defined as SE(estimate)/estimate, expressed as a percentage, with SE(estimate) retrieved directly from the NONMEM output file; V_c_, central volume of distribution; V_ery_, cell volume of distribution; σ_prop_, exponential residual error on plasma (p), erythrocytes (ery), and urine.

### 3.4 In vitro experiments

**Figure 3:**
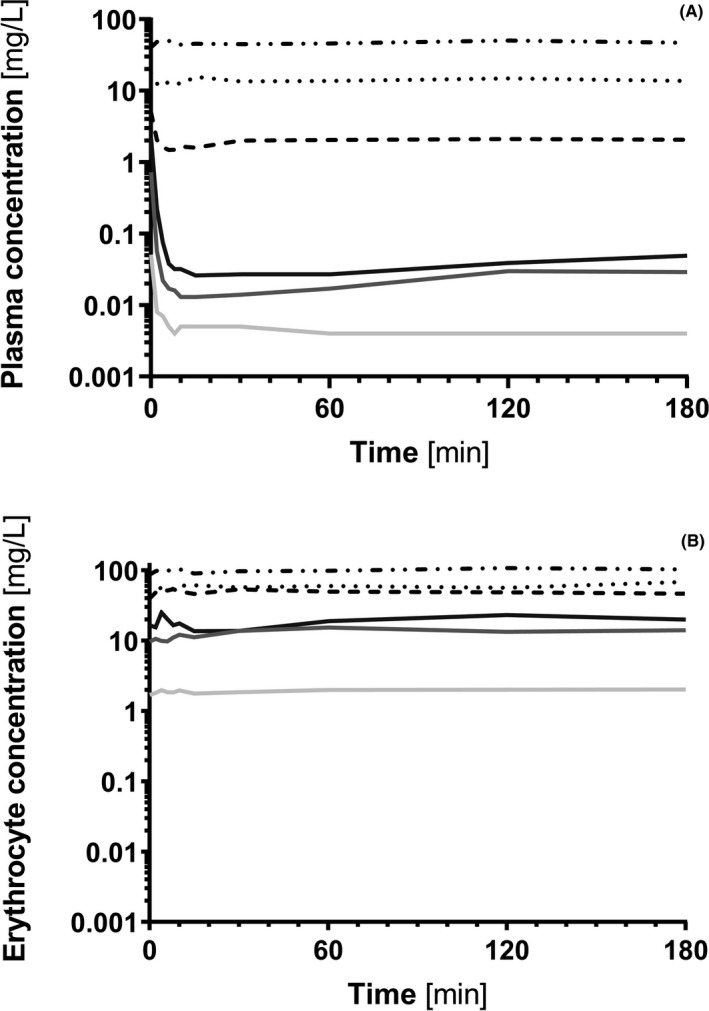
Concentrations of sultiame in plasma (A) and in erythrocytes (B) along incubation time during in vitro spiking experiments at 37°C and with target concentrations of 1 mg/L (light grey), 5 mg/L (dark grey), 10 mg/L (black), 20 mg/L (dashed line), 40 mg/L (dotted line), and 80 mg/L (dashed‐dotted line)

**Figure 4:**
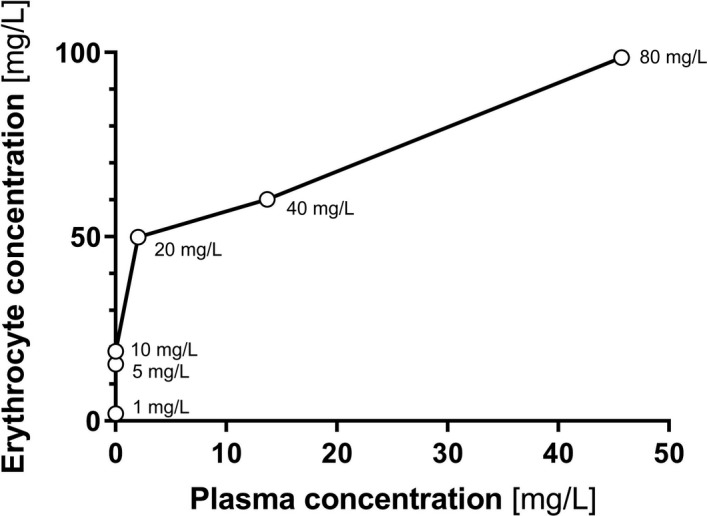
In vitro concentrations of sultiame in erythrocytes versus plasma: values at equilibrium (after 60 minutes of incubation) are represented for each spiking experiments. Open circles represent the spiked concentration of sultiame with the corresponding plasma and erythrocyte concentrations

**Figure 5:**
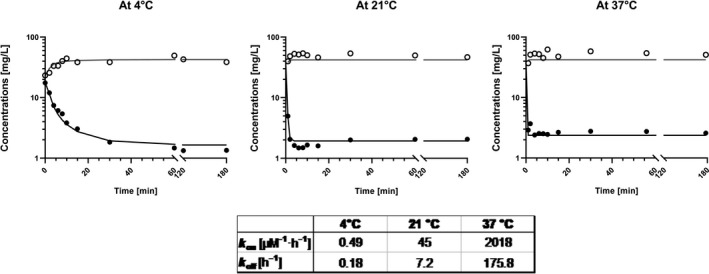
In vitro concentrations of sultiame in erythrocytes and plasma at different temperatures, values of concentrations at, respectively, 4°C, 21°C, and 37°C are represented along incubation time (with a concentration of 20 mg/L). Observed erythrocyte concentrations (open circles), observed plasma concentrations (black circles), and predicted concentration profiles (continuous lines) are represented. Estimates of k_on_ and k_off_ for each temperature derived from the model are represented in the table below

The concentration‐time profiles describing sultiame influx into erythrocytes at different concentrations are shown in Figure 3. Sultiame was taken up almost instantaneously into the erythrocytes and the erythrocyte/plasma ratio reached a steady state after about 10 minutes. This erythrocyte/plasma ratio decreased from 800 at concentrations of 10 mg/L or less, to less than 30 for concentrations of 20 mg/L and above. These results indicate that the saturation of erythrocytes occurs around or slightly above 20 mg/L (of whole blood concentration). At higher concentrations, the excess of sultiame increasingly distributes between the plasma and the erythrocytes (Figure 4). Decreasing the experiment temperature slowed down the rate of entry of sultiame into the cells, without significantly modifying the erythrocyte/plasma ratios. It allowed the estimation of k_on_ and k_off_ using a similar ligand to receptor model at 4°C where the rate of entry was observable. Values at 37°C could be deduced using Arrhenius’ law (Figure 5) and were, respectively, of 2018 µM^−1^ h^−1^ and 175 h^−1^. The efflux experiments revealed the release of only minute amounts of sultiame from incubated erythrocytes into blank plasma (data not shown). Eventually, competition studies indicated that sultiame's affinity for erythrocytes is significantly higher than zonisamide's and topiramate's, both molecules having limited capacity to displace sultiame from its erythrocyte binding sites. Conversely, adding sultiame to a sample enriched with zonisamide led to a clear efflux of zonisamide from erythrocytes, translating into an increase in plasma level. On the other hand, topiramate did not really display affinity for erythrocytes, with an erythrocyte/plasma ratio close to 1, not influenced by the addition of sultiame (data not shown).

## 4 DISCUSSION

To our knowledge, our preliminary clinical PK study is the first to provide a detailed description of sultiame's PK characteristics, since the patenting of this antiepileptic agent in 1955 and its commercialization in 1960.^9^ The PK results obtained after single oral administrations of 50, 100, and 200 mg in four healthy volunteers showed striking nonlinear disposition of sultiame, with a 10‐fold increase in C_max_ for each doubling of dose. Erythrocyte/plasma concentration ratio further confirms sultiame's strong affinity for erythrocytes. The noticeable decrease in this ratio along escalating dosages indicates saturable binding. This nonlinearity is likely to result from the strong affinity of sultiame for CA abundant in erythrocytes.

Indeed, our in vitro spiking experiments confirm that sultiame's distribution into erythrocytes occurs quickly, with a threshold for saturation reached at whole blood concentrations of about 20 mg/L. Limited efflux was observed, confirming a strong affinity; yet a covalent binding of sultiame to intracellular CA is uncertain, considering that a dissociation rate constant (k_D_) of 4.5 µM could be estimated in vitro*.* 15, 16 A value comparable to the estimated k_D_ of our final model (k_off_/k_on_ = 4 µM). Competition studies indicated that sultiame had a stronger affinity for erythrocytes than zonisamide. Clinically, this suggests that displacement of sultiame from the erythrocytes compartment is unlikely to occur with topiramate or zonisamide, while sultiame addition may lead to an at least transient increase in free zonisamide, with a potential risk of increased effects or toxicity. Fluctuations in the plasma/erythrocyte ratio might also be associated with variations in efficacy when sultiame is given as an add‐on antiepileptic drug in clinical practice.

Using noncompartmental approaches, plasma and especially whole blood t_1/2_ appeared to be markedly longer than previously reported.^6^, ^7^, ^8^ The range 2‐16 hours currently reported in the manufacturer's information might have resulted from difficulties to determine low sultiame concentrations with analytical methods available in the past. Considering whole blood, we observed t_1/2_ values of up to 13 days. As the terminal plasma t_1/2_ is dependent of erythrocytes affinity for sultiame and limited by its dissociation from its binding site in erythrocytes, our compartmental model predicts that plasma concentration curves should display a terminal t_1/2_ not different from whole blood. However, plasma concentrations followed up during this terminal phase fall were close to the limit of quantification of method, hence the shorter apparent t_1/2_ observed in our plasma values. Further studies involving the repeated administrations of daily doses might allow a better characterization of the terminal phase of plasma concentrations after treatment interruption. Still an important accumulation of sultiame during long term is not expected despite this presumably long t_1/2_, due to the saturable aspect of the uptake by the receptor‐rich intracellular compartment.

The distribution volume corresponding to whole blood ranges from 6.8 to 16.2 L according to noncompartmental calculations, while it is much larger with respect to plasma, in line with sultiame distribution outside of the plasma compartment, into erythrocytes and others tissues containing CA. Plasma and whole blood noncompartmental V/F and CL/F estimates reveal as well a striking dose dependency, consistent with the saturable aspect of sultiame distribution. Our compartmental model does not indicate that sultiame CL/F from the central plasma compartment would have any nonlinear aspect. In terms of urinary excretion, a constant renal CL/F (range: 0.13‐0.17 L/h) is actually observed, despite the nonlinearity in urinary recovery of unchanged sultiame, again consequent to saturable intracellular uptake. The fraction eliminated by renal route is 28.8%, as already described in the summary of product characteristics.^8^

A two‐compartment model fitted by nonlinear mixed‐effect regression was able to describe adequately our plasma, erythrocytes PK observations with an additional compartment for urine data, assuming a nonlinear distribution and a linear elimination of sultiame. A ligand to receptor saturation model fitted remarkably well with the hypothesized binding of sultiame to erythrocytes, supposedly onto CA abundant in these cells, but also present in other tissues including brain. The plasma CL/F of 10.0 L/h estimated with this model is of the same order of magnitude of values calculated with noncompartmental formulas. Volumes of distribution of 64.8 L for plasma and 3.3 L were estimated for the erythrocyte compartment. As already mentioned, the erythrocyte compartment must be considered to encompass as well other tissues containing CA able to bind sultiame. Yet its volume only moderately exceeds the 2 L or so corresponding to the total erythrocytes mass of normal adults,^17^ which indicates that erythrocytes actually contain the largest part of CA able to bind sultiame in the organism. A maximal binding capacity of 111 mg was estimated, corroborating the saturation of distribution observed in vivo for doses of 100 mg and above (see concentrations in erythrocytes in Figure 2). This estimate is consistent with our in vitro evaluation of a binding capacity slightly exceeding 20 mg per liter of whole blood, considering that the normal volume of whole blood amounts to 4.5‐5 L. A ligand to receptor saturation model actually fitted our data better than the nonlinear disposition models found in the literature that used a sigmoid B_max_ equation to account for nonlinearity.^11^, ^18^ A ligand to receptor model, where the ratio of k_on_/k_off_ accounts for the dissociation constant k_D_ found at the denominator the B_max_ sigmoid equation, probably describes more accurately the disposition of sultiame before steady‐state conditions are reached.

As our population model is based on a limited amount of data, between subject variability and estimation of errors are evaluated with poor precision, as indicated by their wide standard error and CI95%.

Globally, our results have mainly an orientation value and should essentially serve to design further clinical studies with sultiame, particularly in the pediatric population as part of therapy of benign childhood epilepsy with centrotemporal spikes.^3^ They should also be taken into consideration to elaborate an adequate framework of interpretation for the therapeutic monitoring of sultiame circulating concentrations, not only in plasma but also in whole blood, which might present a clinical interest as for other recent antiepileptic agents.^19^

## CONFLICT OF INTEREST

The authors declare that Advicenne Pharma, France, provided financial support for this study. No other conflict of interest is reported.

## AUTHOR'S CONTRIBUTIONS

KD, PT, CB, and TB contributed to the literature search and data collection. KD, PT, TB, LAD, AC, CG, and LAG contributed to study design. LAD, TM, and EC contributed to the development of the validated analytical method. KD, MG, SL, and TB participated in data analysis and interpretation. KD wrote the manuscript and PT, LAD, EC, MG, SL, CG, AC, and TB critically reviewed the manuscript.

## ETHICAL STATEMENT

All procedures performed in studies involving human participants were in accordance with the ethical standards of the competent research ethics committee and regulatory authorities and with the 1964 Helsinki Declaration and its later amendments. Informed consent was obtained from all individual participants involved in the study.

## Data Availability

The data that support the findings of this study are available in DDMoRe Repository at http://repository.ddmore.foundation//, reference number DDMODEL00000298. These data were derived from real data and key dosage and concentration data were not altered.
