# Supplementary figures and images for: Sultiame pharmacokinetic profile in plasma and erythrocytes after single oral doses: A pilot study in healthy volunteers
Source: Pharmacol Res Perspect. 2020 Jan 28;8(1):e00558. doi: 10.1002/prp2.558 (PMC6986439; doi:10.1002/prp2.558)

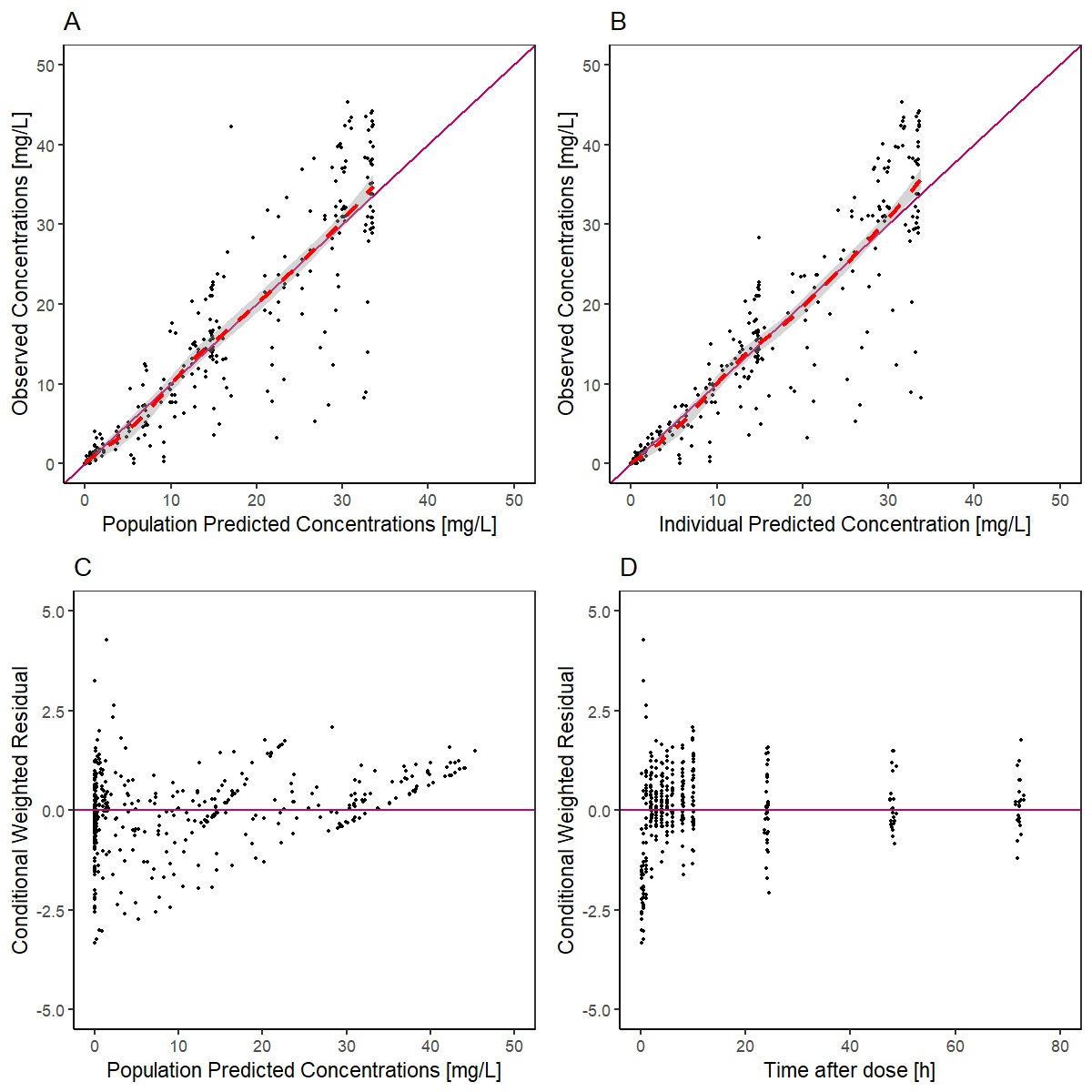

Supplement: Supplementary file 1 [file PRP2-8-e00558-s001.jpg]

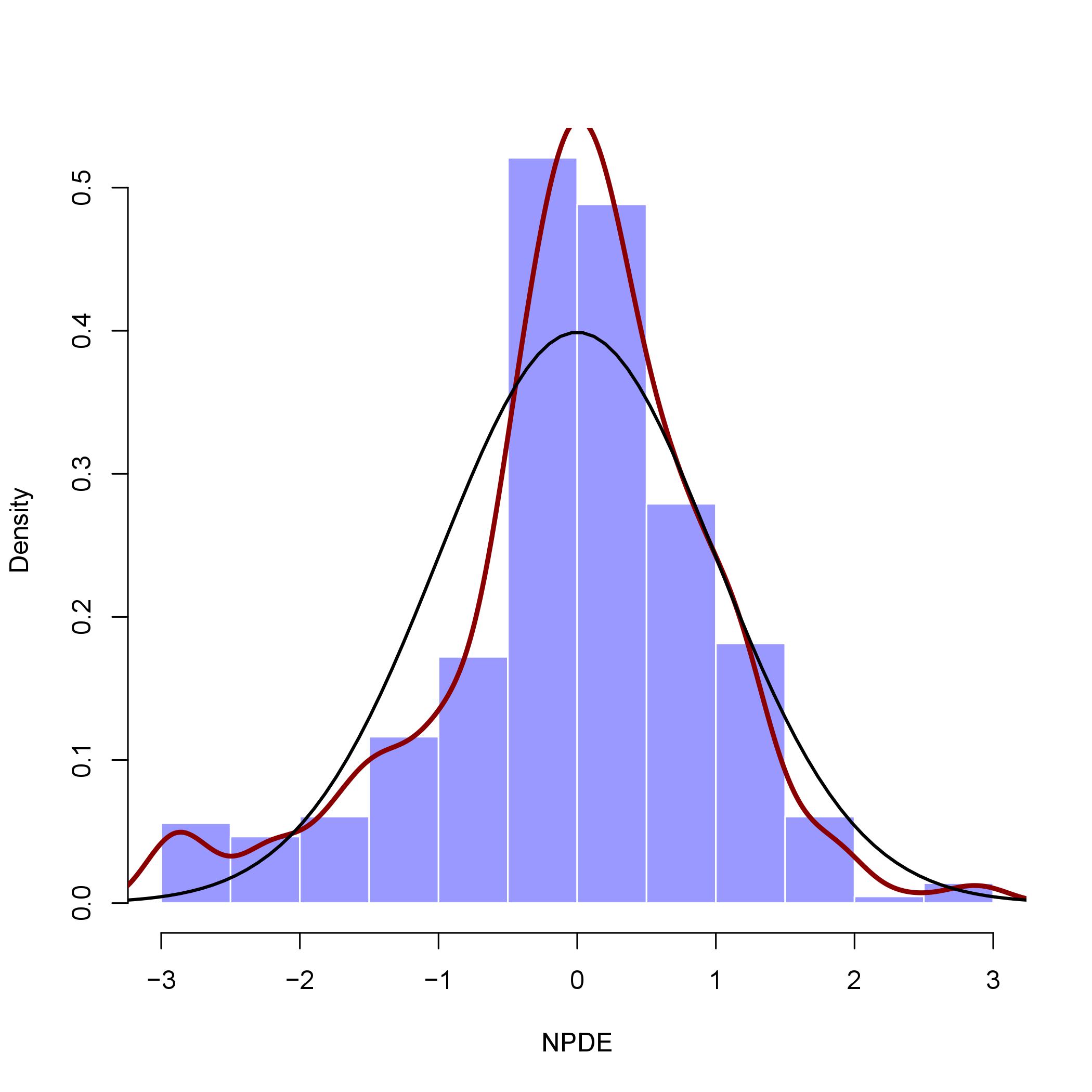

Supplement: Supplementary file 2 [file PRP2-8-e00558-s002.jpg]

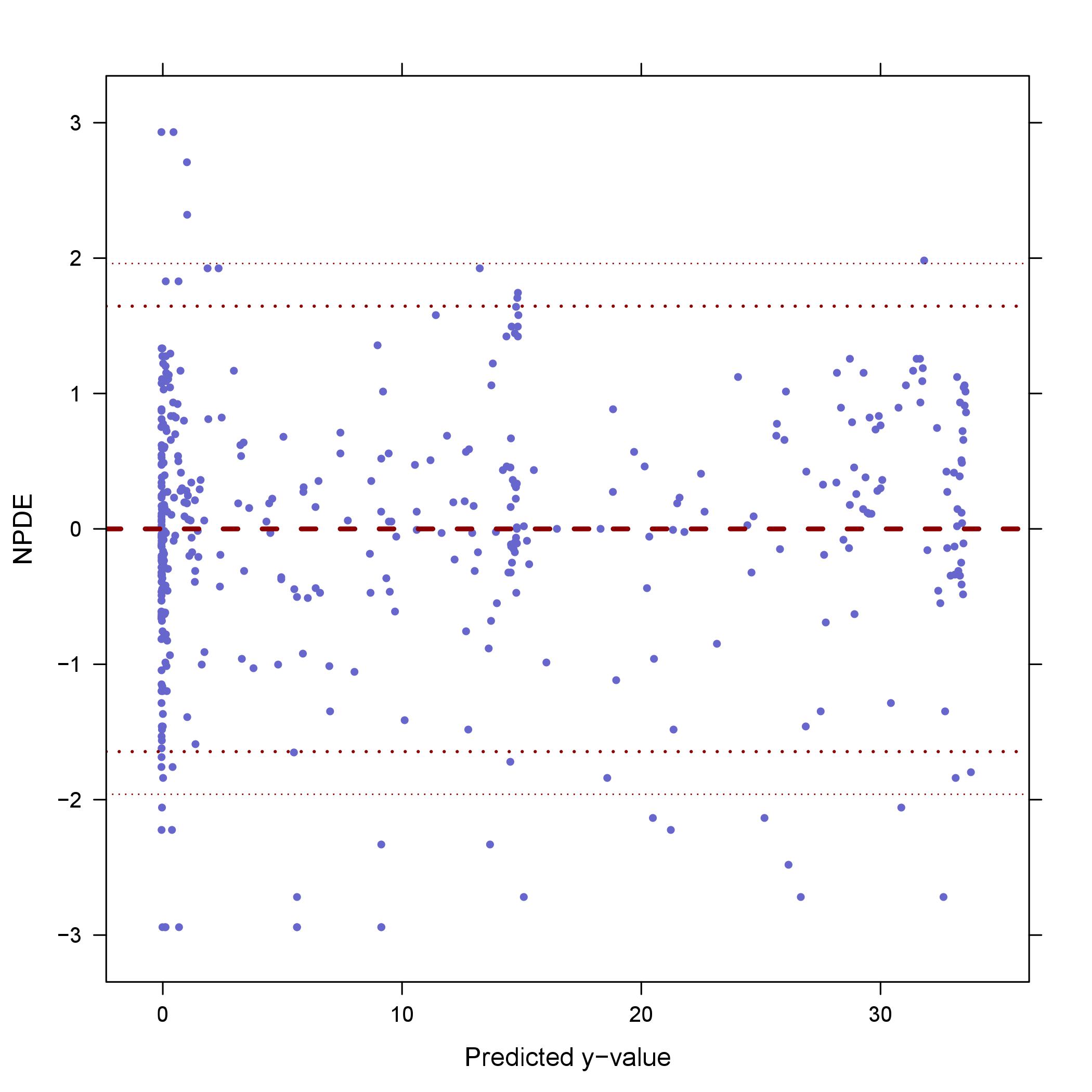

Supplement: Supplementary file 3 [file PRP2-8-e00558-s003.jpg]

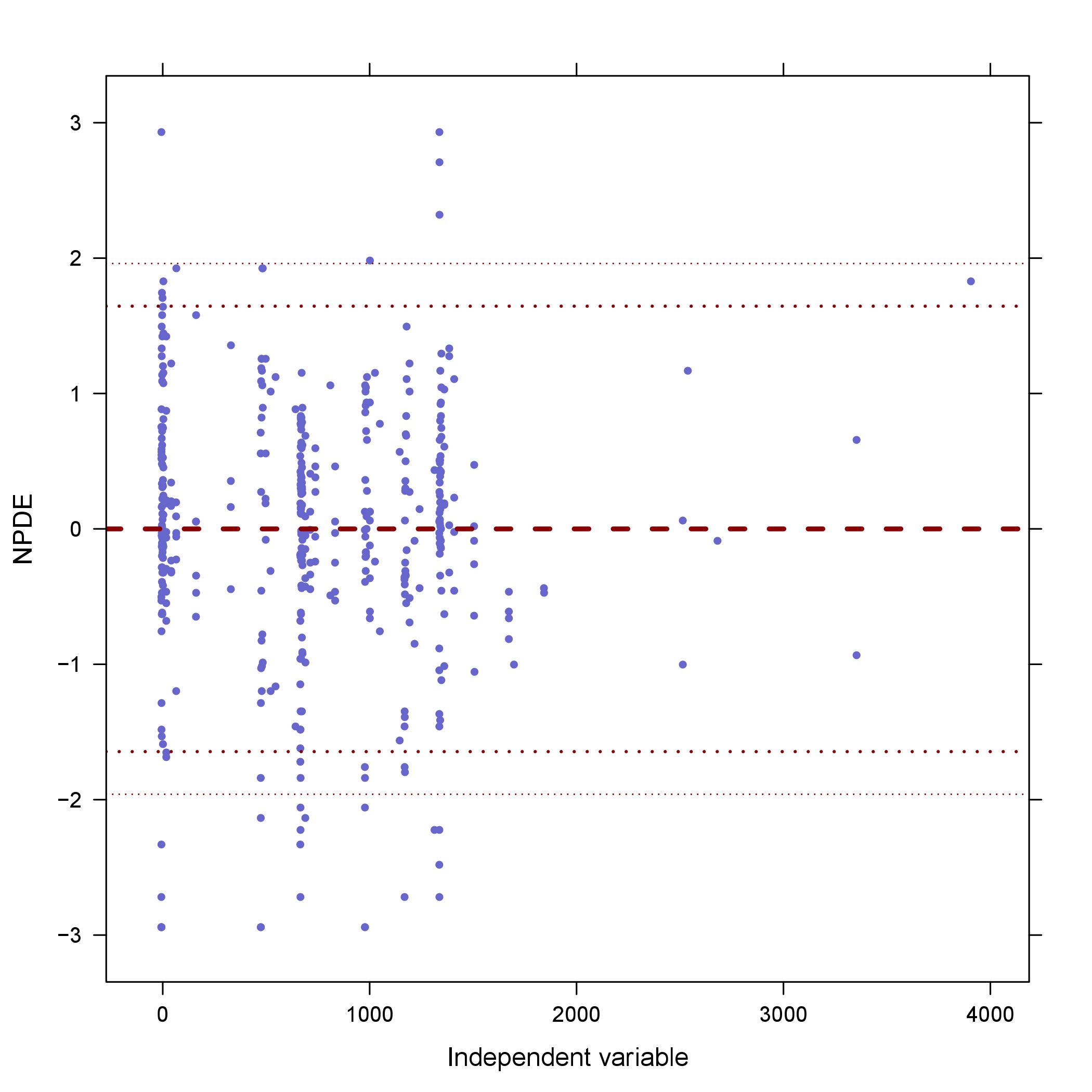

Supplement: Supplementary file 4 [file PRP2-8-e00558-s004.jpg]

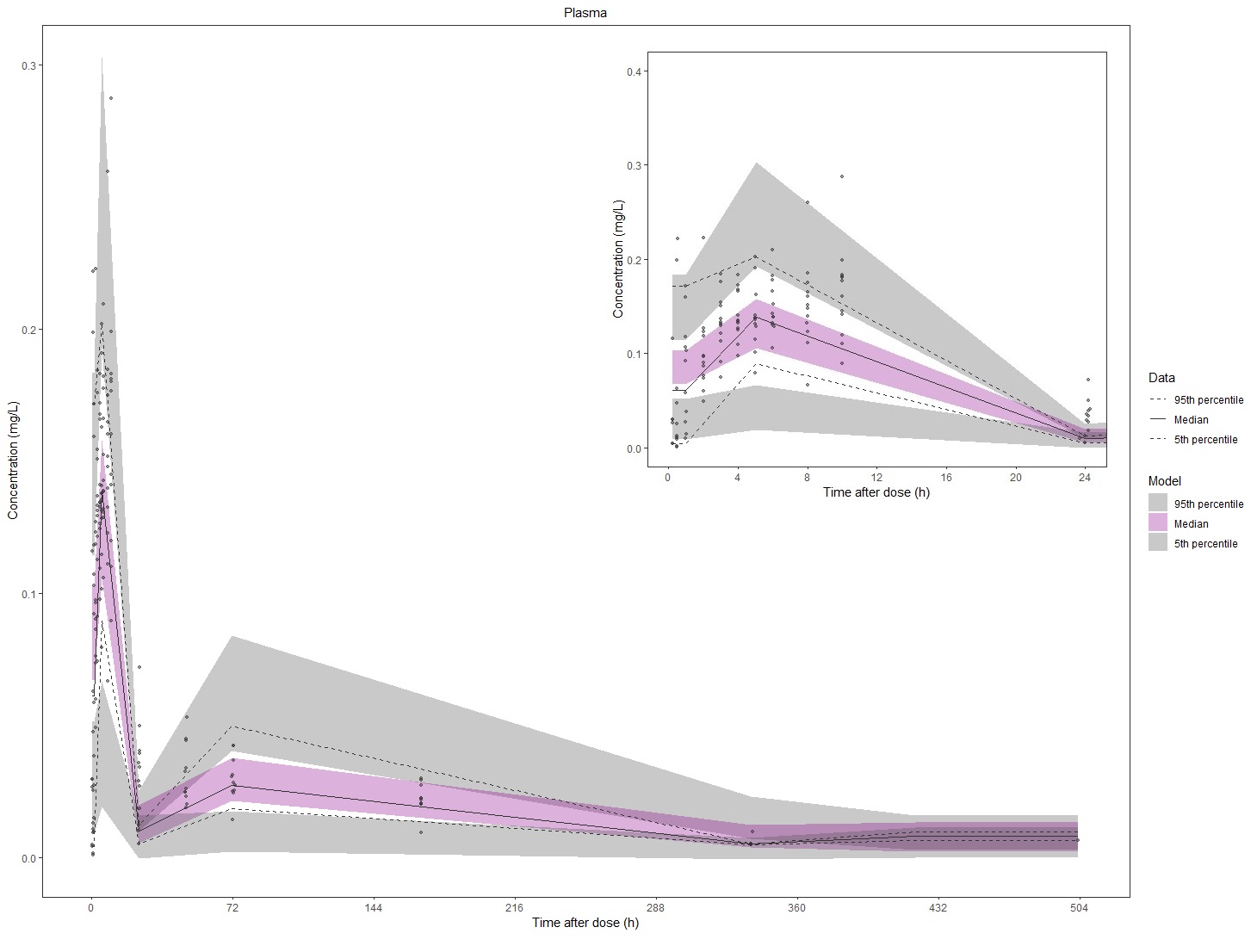

Supplement: Supplementary file 5 [file PRP2-8-e00558-s005.jpg]

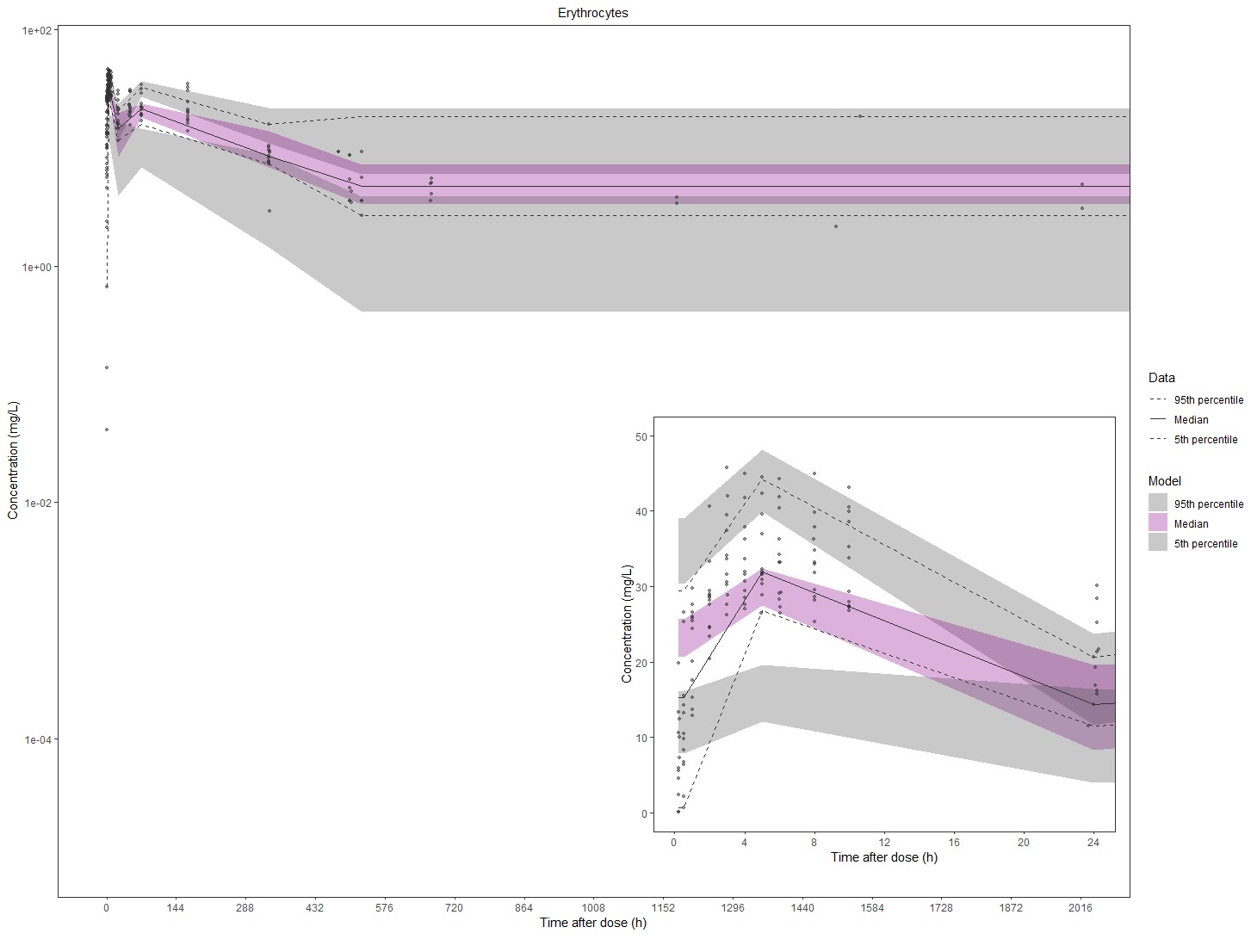

Supplement: Supplementary file 6 [file PRP2-8-e00558-s006.jpg]

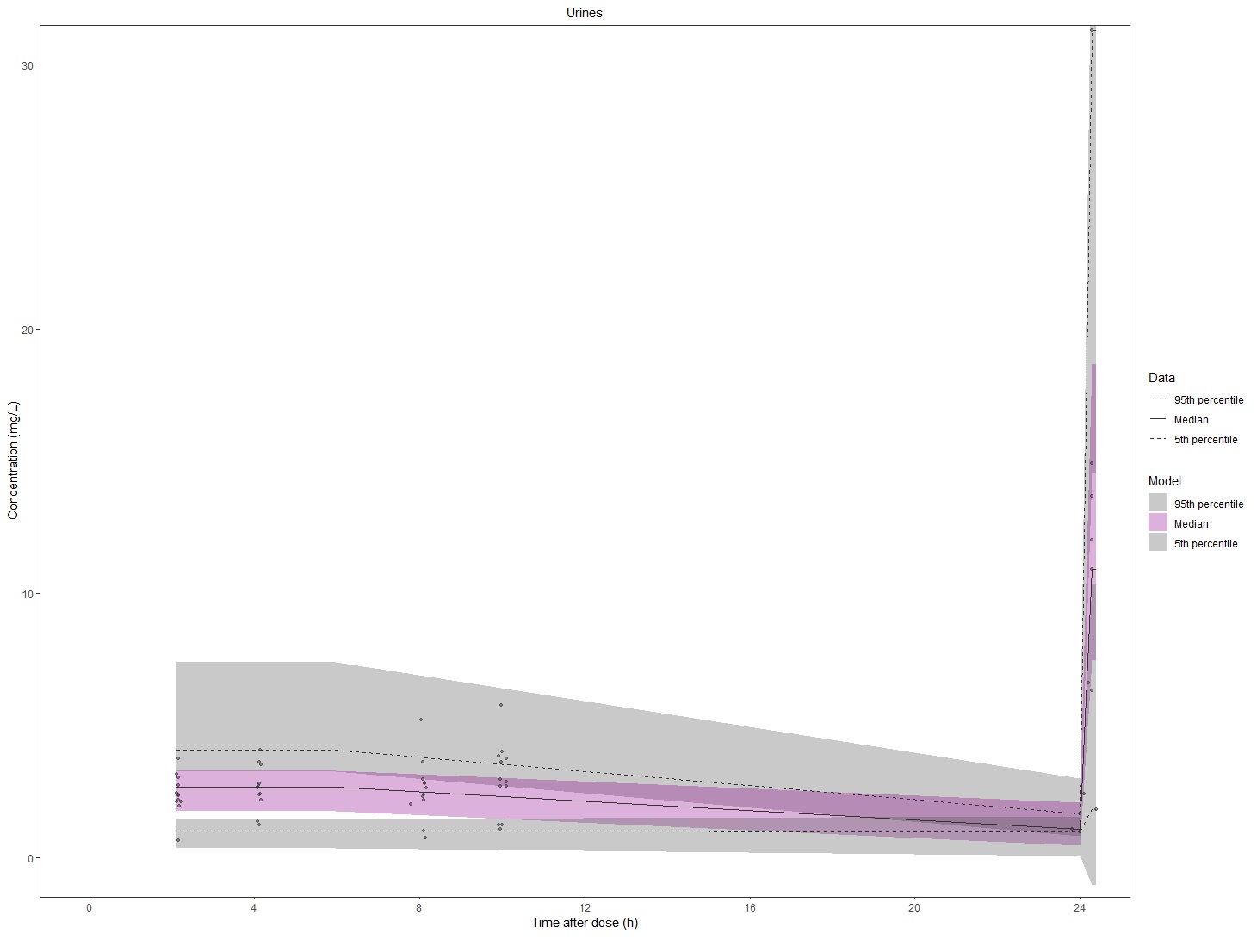

Supplement: Supplementary file 7 [file PRP2-8-e00558-s007.jpg]

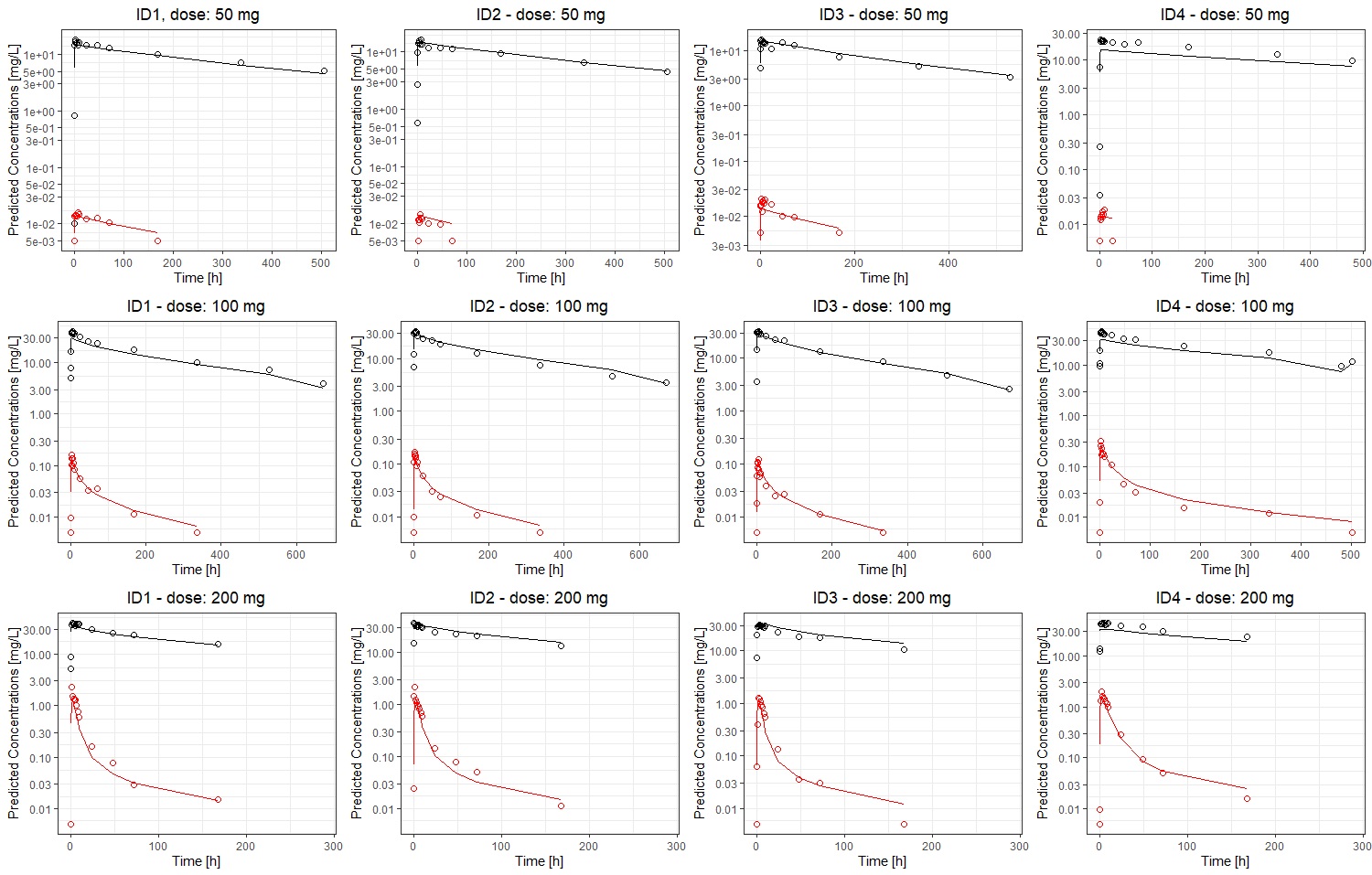

Supplement: Supplementary file 8 [file PRP2-8-e00558-s008.jpg]
