# Supplementary material for: Sultiame pharmacokinetic profile in plasma and erythrocytes after single oral doses: A pilot study in healthy volunteers
Source: Pharmacol Res Perspect. 2020 Jan 28;8(1):e00558. doi: 10.1002/prp2.558 (PMC6986439; doi:10.1002/prp2.558)
Supplement: Supplementary file 9 [file PRP2-8-e00558-s009.docx]

Sultiame Pharmacokinetic Profile in Plasma and Erythrocytes after Single Oral Doses: A Pilot Study in Healthy Volunteers

Kim Dao^1^, Paul Thoueille^1^, Laurent Arthur Decosterd^1^, Thomas Mercier^1^, Monia Guidi^1,2^, Carine Bardinet^1^, Sébastien Lebon^3^, Eva Choong^1^, Arnaud Castang^4^, Catherine Guittet^4^, Luc-André Granier^4^, Thierry Buclin^1^

1. Service of Clinical Pharmacology, Lausanne University Hospital, and University of Lausanne, Switzerland.
2. School of Pharmaceutical Sciences, Institute of Pharmaceutical Sciences of western Switzerland, Geneva, Switzerland
3. Paediatric Neurology Unit, Department Mother-Woman-Child, Lausanne University Hospital, and University of Lausanne, Lausanne, Switzerland.
4. Advicenne Pharma SA, Nîmes, France

Methods

**Population pharmacokinetic model**

Our population PK analysis used a non-linear mixed effect two-compartment model with first-order absorption incorporating a saturable ligand to receptor binding. We fitted it to our data with the NONMEM software (version 7.4, ICON Development Solutions, Hanover MD, USA), using the ADVAN13 subroutine.

Differential equations were written to account for the evolution of sultiame amounts (in mg):

- at the absorption site (A_a_);
- in the plasma (A_p_, assumed to be in instantaneous equilibrium with other body fluids where free sultiame distributes);
- bound to erythrocytes receptors (A_ery_, assumed to encompass as well sultiame bound to receptors contained in other cells);

A renal outflow (A_ren_) was eventually added to accommodate sultiame urinary data.

The following eight parameters were needed to describe the model:

- first-order rate constant for absorption (k_a_);
- systemic total plasma clearance (CL/F);
- association rate constant onto receptors (k_on_);
- dissociation rate constant from receptors (k_off_);
- maximal specific binding capacity of receptors (B_tot_);
- central volume of distribution (V_c_/F);
- erythrocytes’ volume of distribution (V_ery_);
- renal fraction of total clearance (Q_ren_).

It is worth noting that V_ery_ actually includes the volume of other types of cells containing receptors, assumed to behave in a similar way to erythrocytes. CL/F and both V_c_/F and V_ery_ are apparent parameters as the oral bioavailability could not be estimated. The first order elimination rate constant from the central compartment was calculated as k_e_ = (CL/F)/(V_c_/F) and used in the differential equations below.

The evolution over time of the amount A_a_ of sultiame remaining in the absorption compartment was described according to the differential equation:

$\frac{{dA}_{a}}{dt}= - k_{a}\cdot A_{a}$ with A_a_ = Dose at t = 0 (1)

The evolution over time of the amount A_p_ of sultiame in the central compartment, reflected in plasma concentration, was described according to the differential equation:

$\frac{{dA}_{p}}{dt}= k_{a}\cdot A_{a}- k_{e}\cdot A_{p}-k_{on}\cdot A_{p}\cdot\left( B_{tot}-A_{ery} \right)+ k_{off}\cdot A_{ery}$ with A_p_ = 0 at t = 0 (2)

The evolution over time of the amount A_ery_ of sultiame bound to receptors in the cell compartment was described over time according to the differential equation:

$\frac{{dA}_{ery}}{dt}= k_{on}\cdot A_{p}\cdot\left( B_{tot}-A_{ery} \right)- k_{off}\cdot A_{ery}$ with A_ery_ = 0 at t = 0 (3)

Notice that A_ery_ simultaneously expresses the amount of binding sites occupied by sultiame (B_bound_). Thus, the amount of free binding sites corresponds to B_free_ = (B_tot_ – A_ery_), and the model only needs the parameter B_tot_ to characterize the size of the saturable compartment associated to receptor binding (in mg of sultiame). The values of k_on_ and k_off_ characterized *in vitro* at 37°C were used in the *in vivo* model as initial estimates, and the k_on_ was fixed to 2018 µM^-1^· h^-1^.

Finally, the cumulating amount A_u_ of sultiame (mg) excreted in urine over time was described according to the differential equation:

$\frac{{dA}_{u}}{dt}= k_{e}\cdot A_{p}\cdot Q_{ren}$ with A_u_ = 0 at t = 0 (4)

The model was fitted to the data using first order conditional estimation (FOCE) to obtain values for the eight model parameters listed above. Alternate models were tried, for example a sigmoid B_max_ model, [1, 2] a model with a peripheral distribution compartment, a model with an additional intracellular compartment for free sultiame, assuming cell entry and exit rate constants distinct from k_on_ and k_off_, respectively. None of these models provided a better description of the data. The comparison of models was based on the difference of objective function value (OFV), Akaike criterion (AIC) and visual goodness-of-fit graphics. Considering an approximated chi-square distribution of the OFV, a statistical difference of OFV > 10.83 points and AIC of > 6 (p < 0.001) were considered significant. Various combinations of inter-individual variability on PK parameters were tested, the best one corresponding to variability set onto CL/F and Q_ren_. Different residual errors were assumed for plasma, erythrocyte and urine samples. A proportional error was appropriate for plasma, erythrocyte and urine values. Due to the small number of study subjects, no demographic covariates were tested for inclusion in the model.

**Internal model validation**

The stability of the final model was assessed by means of the bootstrap method implemented in Perl speaks NONMEM (PsN, version 4.8.1). [3] Median parameters values with their 95% confidence interval (CI95%) were derived from 2000 replicates of the initial dataset and compared with the original estimates. Goodness-of-fit plots were used for model validation (**Figure 6**). The predictive performance of the pharmacokinetic model was evaluated by calculation of normalized prediction distribution errors (NPDEs), simulating each original observation 3000 times. The NPDEs and their distributions were then computed (**Figure 7**). Prediction-corrected visual predictive checks (pcVPC) were also performed using PsN-Toolkit and Xpose4 (version 4.3.5, Uppsala, Sweden) [4] by simulations based on the final PK estimates using 1000 individuals to calculate median concentration-time profile and 95% prediction intervals (PI95%) (**Figure 8**). Accuracy and precision of the model were estimated through mean prediction error (MPE) and root mean squared error (RMSE) using log-transformed concentrations. [5]

Results


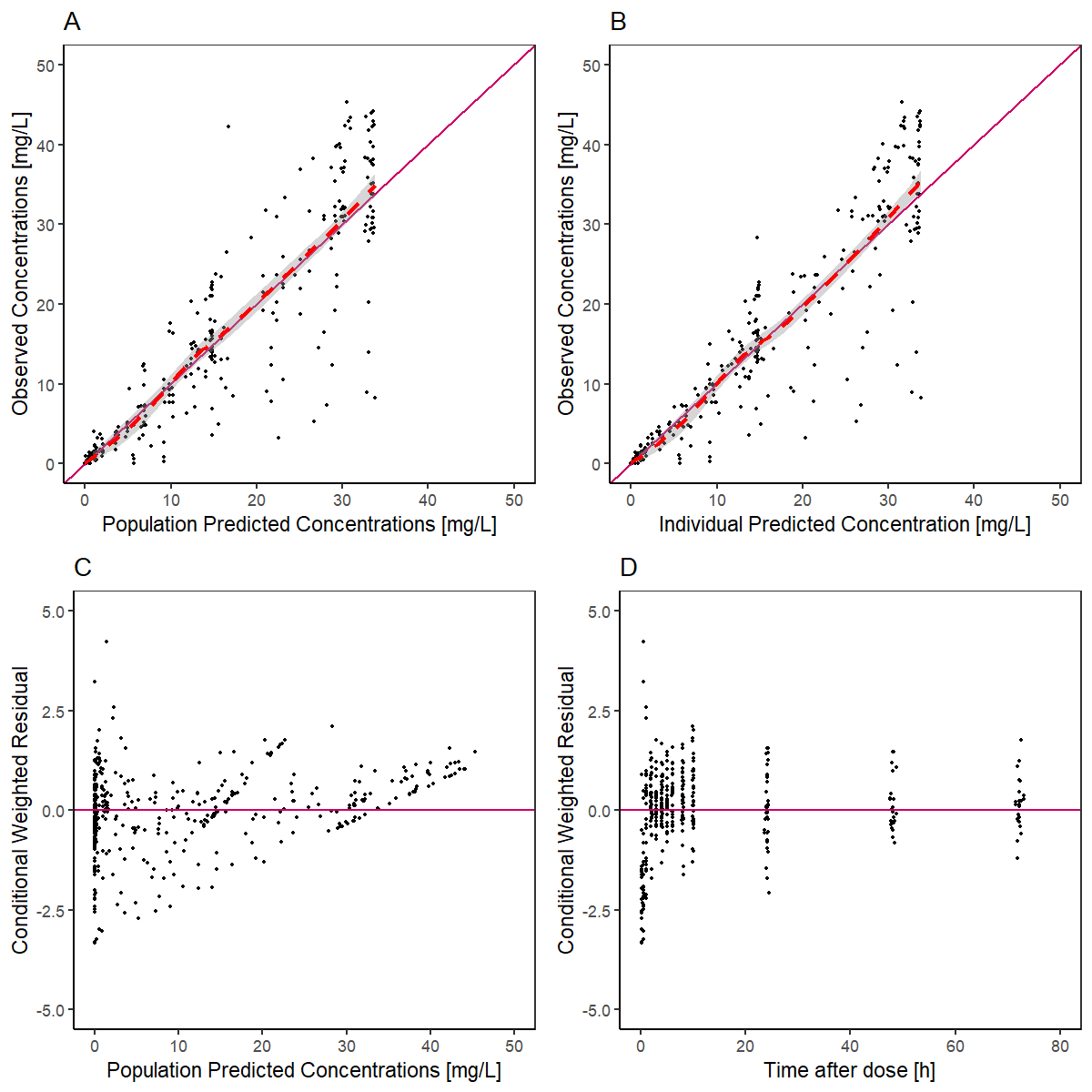


**Figure 6: Goodness of fit plots of the final PK model**: observed concentrations versus population predicted concentrations (A), versus individual predicted concentration (B), conditional weighted residual versus population predicted concentrations (C), versus time after dose (D)


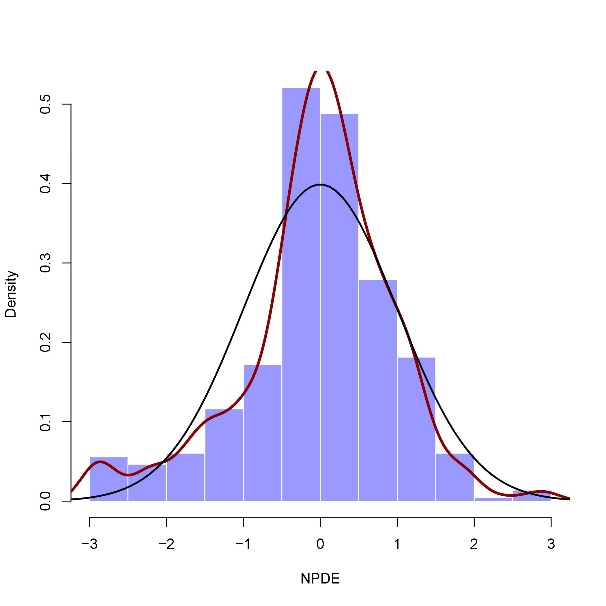
(a)


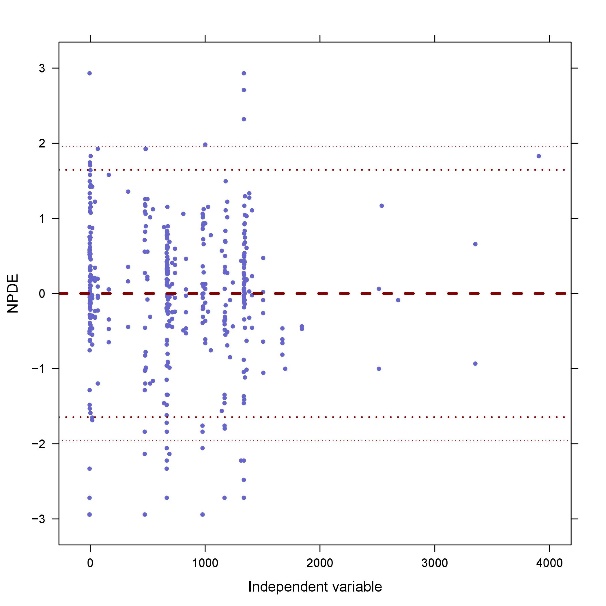
(b)


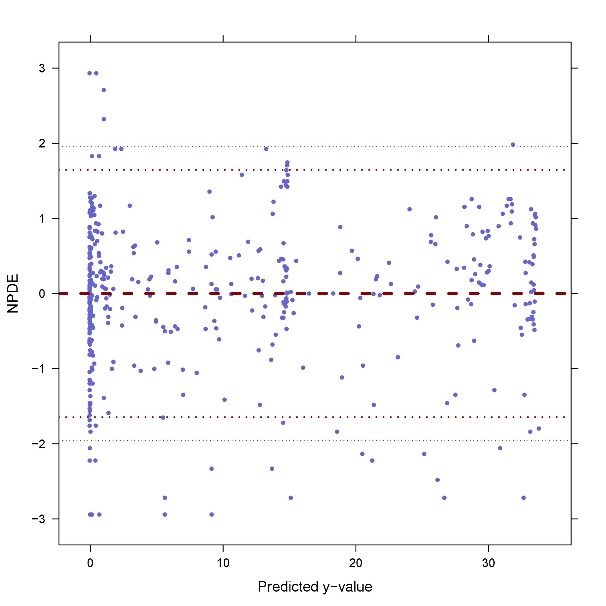
(c)

**Figure 7: Normalized prediction error (NPDE):** (a) distribution of NPDE, (b) NPDE versus time and (c) NPDE versus predicted concentrations.


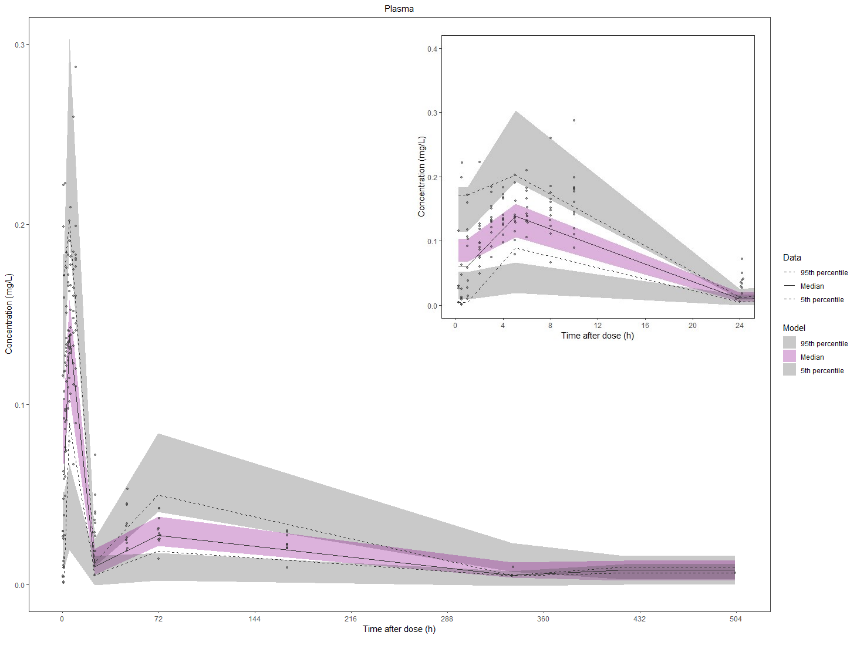
(a)
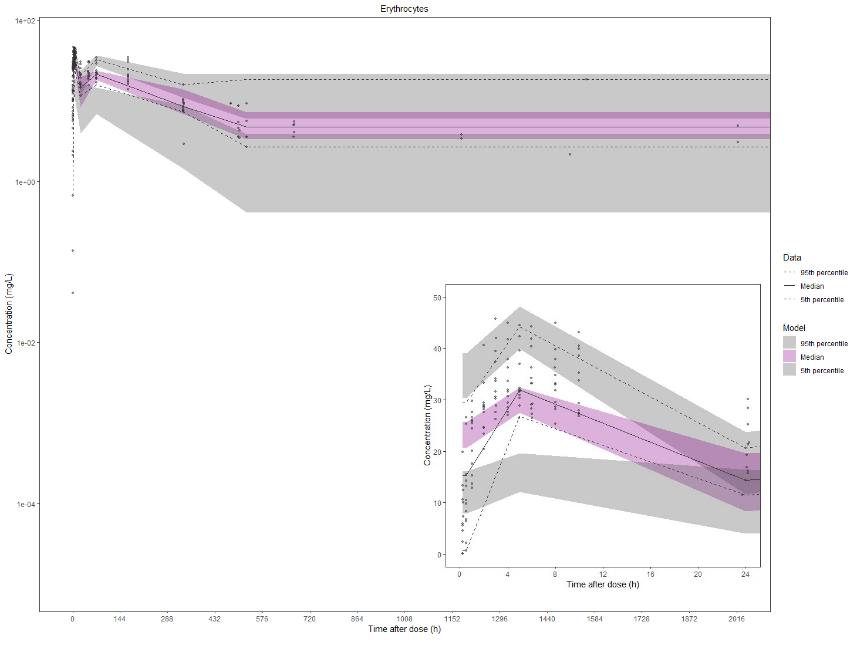
(b)
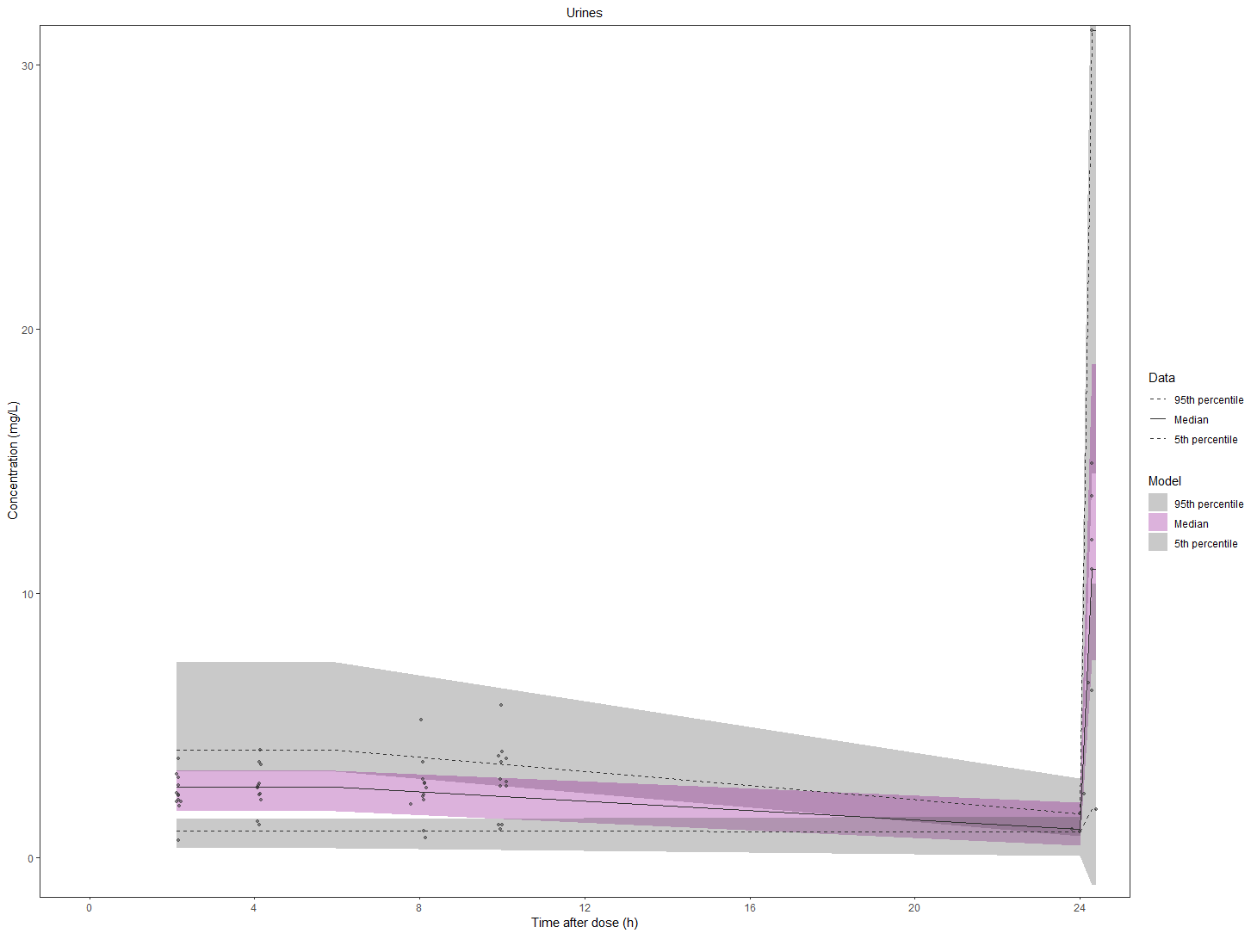
(c)

**Figure 8:** **Prediction-corrected Visual Predictive Check** of the final model with sultiame plasma (a), erythrocyte (b) and urine (c) prediction-corrected concentrations (circles) and median prediction corrected concentrations (solid line) and 90% confidence interval (semi-solid line). Grey fields represent the model-based percentile 90% confidence interval.

Internal model validation

The model appears to overestimate early concentrations and underestimate the late concentrations in plasma and erythrocytes. These model misspecifications may be related to the paucity of measurements in the absorption phase, the limited number of subjects included and the number of parameters of the model. The conditional weighted residuals show a discrete upward bias but are mostly within the recommended +/-3 range.

**PK model**


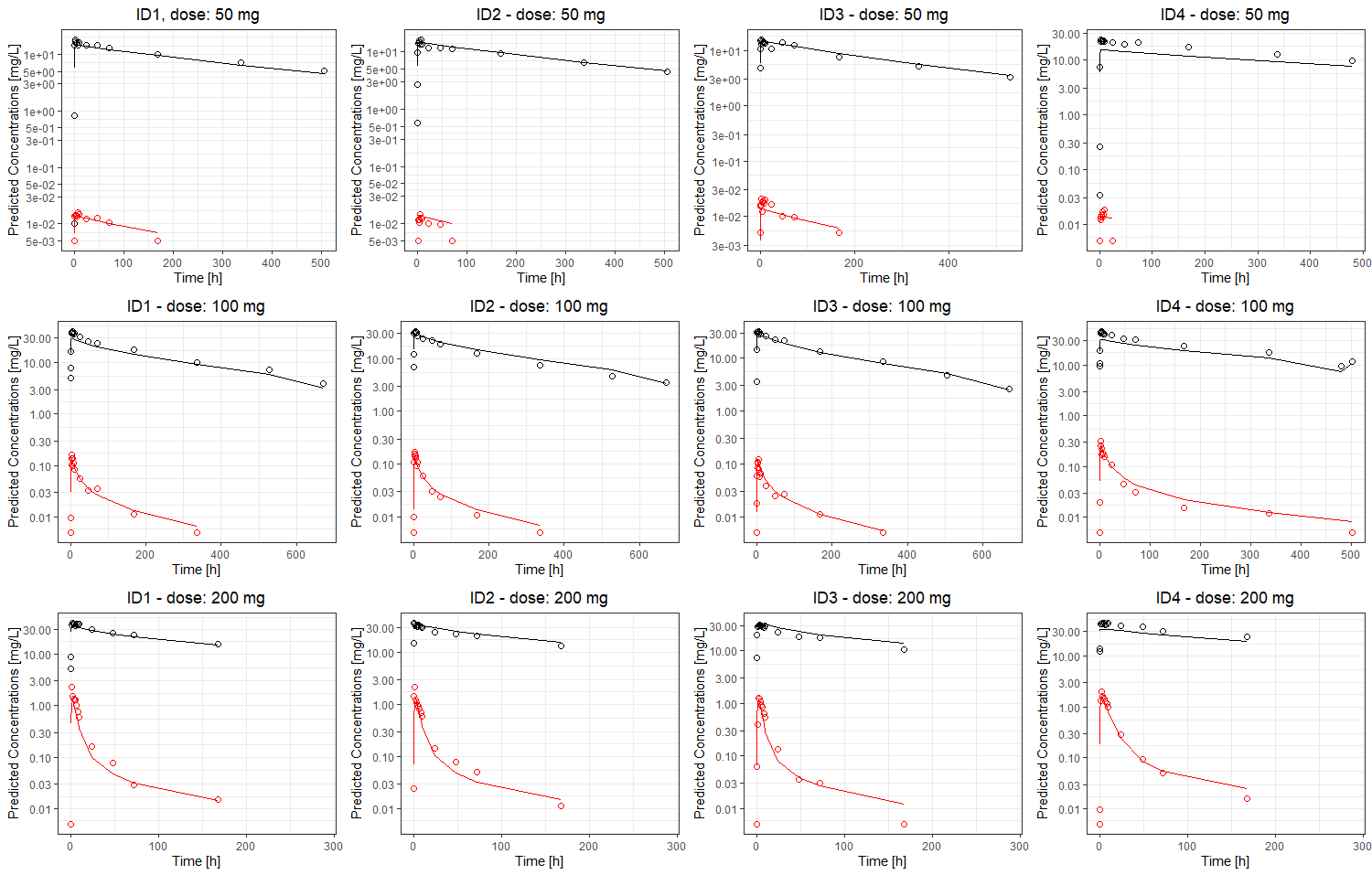


**Figure 9:** Individual profiles of the two-compartment model describing sultiame concentrations in plasma (red) and erythrocytes (black) according to time. Observations are represented by open circles, predictions by a continuous line.

**References**

1. Snoeck, E., et al., *The implications of non-linear red blood cell partitioning for the pharmacokinetics and pharmacodynamics of the nucleoside transport inhibitor draflazine.* Br J Clin Pharmacol, 1996. **42**(5): p. 605-13.

2. Zandvliet, A.S., et al., *A semi-physiological population pharmacokinetic model describing the non-linear disposition of indisulam.* J Pharmacokinet Pharmacodyn, 2006. **33**(5): p. 543-70.

3. Lindbom, L., P. Pihlgren, and E.N. Jonsson, *PsN-Toolkit--a collection of computer intensive statistical methods for non-linear mixed effect modeling using NONMEM.* Comput Methods Programs Biomed, 2005. **79**(3): p. 241-57.

4. Jonsson, E.N. and M.O. Karlsson, *Xpose--an S-PLUS based population pharmacokinetic/pharmacodynamic model building aid for NONMEM.* Comput Methods Programs Biomed, 1999. **58**(1): p. 51-64.

5. Sheiner, L.B. and S.L. Beal, *Some suggestions for measuring predictive performance.* J Pharmacokinet Biopharm, 1981. **9**(4): p. 503-12.
